# Identifying and Characterizing Key Nodes among Communities Based on Electrical-Circuit Networks

**DOI:** 10.1371/journal.pone.0097021

**Published:** 2014-06-04

**Authors:** Fenghui Zhu, Wenxu Wang, Zengru Di, Ying Fan

**Affiliations:** School of Systems Science, Beijing Normal University, Beijing, China; Universiteit Gent, Belgium

## Abstract

Complex networks with community structures are ubiquitous in the real world. Despite many approaches developed for detecting communities, we continue to lack tools for identifying overlapping and bridging nodes that play crucial roles in the interactions and communications among communities in complex networks. Here we develop an algorithm based on the local flow conservation to effectively and efficiently identify and distinguish the two types of nodes. Our method is applicable in both undirected and directed networks without a priori knowledge of the community structure. Our method bypasses the extremely challenging problem of partitioning communities in the presence of overlapping nodes that may belong to multiple communities. Due to the fact that overlapping and bridging nodes are of paramount importance in maintaining the function of many social and biological networks, our tools open new avenues towards understanding and controlling real complex networks with communities accompanied with the key nodes.

## Introduction

Many real networks typically contain components in which the nodes are of much denser connections to each other than to the rest of the network. The sets of such nodes are usually called communities or modules [1]–[3]. Communities indicate the existence of different groups that perform specific roles in social and biological networks. Exploring network communities is an important task in the sense that they provide graphical clues to the specific functions of groups of nodes and allows us to explore a network at a coarse level, which is much more helpful for understanding dynamical processes taking place on a network rather than inspect a network as a whole without any a priori knowledge about the similarity and functions of nodes [4]. Thus many methods have been developed for community detection, such as progressively removing the edges with maximum betweenness [5], optimizing the strength of the community by merging nodes [6], the extremal optimization method [7], and approaches based on the dynamical processes taking place on networks [8].

**Figure 1:**
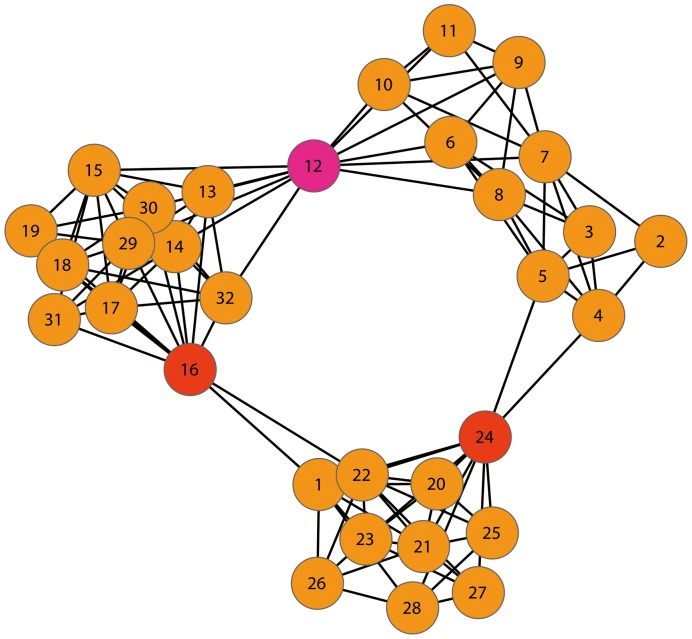
Schematic network composed of 32 nodes and separated into 3 parts. Certain nodes connect the separate parts.

Despite the algorithms developed for detecting communities in complex networks, precisely partitioning communities in many real scenarios is still a challenging problem because of the existence of special nodes that belong to different communities simultaneously, namely, overlapping nodes. Some approaches have been presented attempting to solve the community detection problem associated with overlapping nodes. For example, Palla *et al.* proposed a method based on clique percolation [9]. A community is defined by a set of nodes that can be visited by rolling a *k* clique over the network through other cliques with 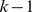 common nodes. Lancichinetti *et al.* proposed an algorithm to detect overlapping and hierarchical structures using a fitness function [10]. In contrast, fuzzy modularity concentrated on the probabilities of each node belonging to different modules [11]. Guimera *et al.* classified nodes based on their roles within communities, using their within-module degree and their participation coefficient to reflect their positions in their own module and with respect to other modules [12]. Nonetheless, to the best of our knowledge, we still lack an efficient method to identify “connectors” among communities without relying on accurate partition of communities. Here we classify connectors into two categories: overlapping node and bridging node. Overlapping nodes refer to the nodes that belong to two or more communities with a number of edges connecting to each community, e.g., node 12 in Fig. 1. Whereas bridging nodes refer to the nodes that belong to a single community but has a few connections to the other communities; in other words, their edges bridge their own communities and the others, e.g. node 16 and 24 in Fig. 1. The two types of nodes play key roles in the communications and interactions among different communities and server as “messengers”. Although we may find the two types of nodes in terms of partitioning communities by using the established methods, it is computational exhausted and considerably depends on the accuracy of detecting communities that has yet not been fully resolved. Despite some interesting methods based on synchronization processes to locate overlapping nodes [8], they are not available for bridging nodes. Moreover, algorithms and tools for tackling overlapping communities in directed networks are still lacking.

In this paper, we propose a method to detect and distinguish overlapping nodes and bridging nodes based on the current flow in the electrical circuits. The current-flow-based methodology has been exploited for studying complex networks, for instance, for exploring transportation dynamics of resistor networks [13] and modeling information flow in biological networks and finding nodes with significant biological function [14]–[17]. Inspired by the insightful approaches, we map an arbitrary network into an electrical-circuit network in which all the edges are resistors with a specific electrical conductance, and a pair of nodes can be set as the source and sink (target) of the current flowing through the network. By combining Kirchhoff's law and Ohm's law, we can calculate the flow of each edge for a given source and target of flow. It is intuitive that overlapping and bridging nodes usually have high current flows as measured by the current-flow centrality *C*, because of their specific positions. Thus the two types of nodes can be distinguished from the other nodes by their high values of *C*. Meanwhile, we offer an imbalance index *D* that captures the imbalance of current flows along the edges of nodes to separate the two types of nodes. In particular, the bridging edges of bridging nodes are of much high current flows than the rest of their edges. In contrast, the current flows along the edges of an overlapping node are relatively balanced because of the fact that the current flow passing through it is shared by its edges densely connecting to both communities. The main advantage of our method is that overlapping and bridging nodes can be identified without knowing the exact community partition of the network, accounting for its high efficiency and feasibility in detecting the key nodes. Moreover, our method can be applied to directed networks in a similar fashion. We substantiate our method in terms of a number of model and empirical networks, including the Lancichinetti-Fortunato-Radicchi (LFR) benchmark with tunable community structure and a power-law degree distribution [18], Zachary's Karate Club (ZK) network [19], the scientific collaboration network in Santa Fe Institute (SFI) [20] and the neural network of C. elegans [21]. The two types of nodes in all the networks are detected with high probability and efficiency. We finally discussed the shortage of our method rooted in the implicit definition of communities.

## Methods

### Electrical-circuit method for undirected and directed network

**Figure 2:**
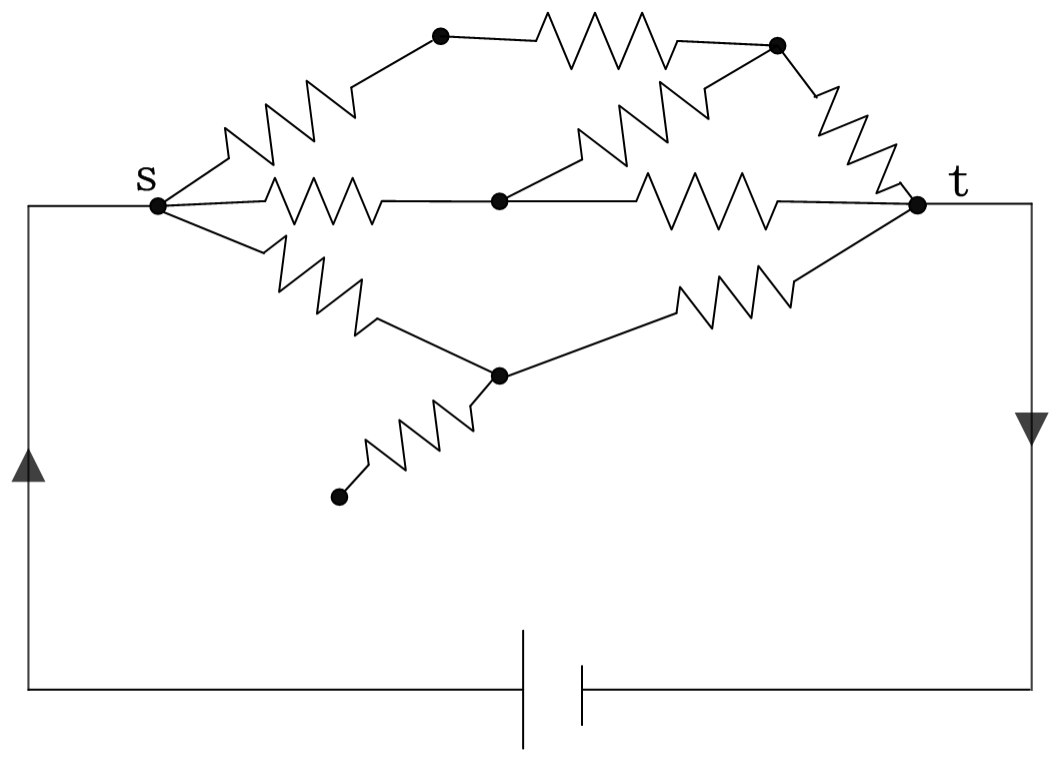
An electrical-circuit network with an electrical conductance on each edge. Current flows through the network from the source node *s* and leaves at the target node *t*, each edge has a fixed conductance.

In an electrical-circuit network generated by placing a resistor with a specific electrical conductance on each edge of the network [22], as shown in Fig. 2, a given pair of nodes will serve as the source and target nodes, where current is injected into the network at the source node and leaves at the target node. In fact, any arbitrary network can be represented as a resistor network.

Current flows from the source node *s* to the target node *t*, which causes a voltage difference between node *s* and node *t*. Ohm' s law states that the current through a conductor between two points is directly proportional to the potential difference across the two points; thus, for a given source-target pair, the current flowing through an arbitrary edge(*i*, *j*) is
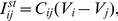
(1)where 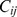 is an element of the adjacency matrix, and *I* represents the current between nodes 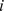 and 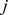 when the current is injected into the source node 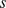 and leaves at the target node 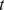.

We consider the general case: node 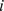 connects to 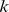 neighbors, and for an arbitrary node 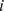, Kirchhoff's law states that the total current flow into or out of any node is zero. Combining Kirchhoff' s law with Ohm's law, implies that the voltages satisfy the equation
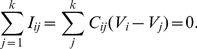
(2)


Physically, the source node maintains a constant potential,and the target is chosen to be the preferred node by which it connects to the ground. For a network with 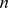 nodes, there are 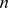 linear equations, which can be written as follows:
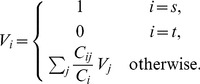
(3)where 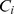 the sum is over all neighbors of node 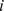. The potential of each node 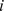 can be solved using an iterative method such as the Jacobi method.

**Figure 3:**
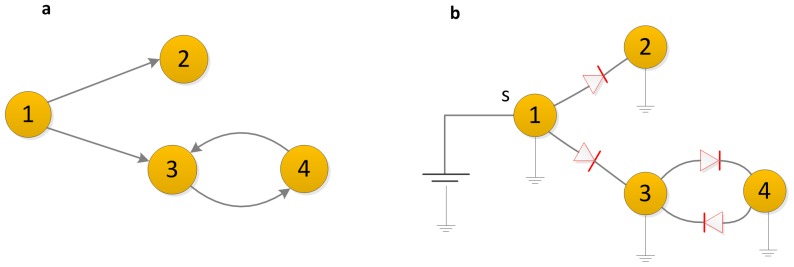
A simple network with four nodes and its equivalent circuit network. Edges are represented by electrical-circuit diodes and nodes are connected to a universal sink.

The method can be extended to a directed network as long as we replace the resistors with an electrical circuit of diodes, as shown in Fig. 3, in this equivalent electrical circuit, all the nodes are connected to a universal sink (ground) whose potential value is zero [23]. The voltages of the nodes need be adjusted to satisfy Kirchhoff's law, which states that the sum of all currents entering node 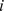 must be equal to the sum of all currents leaving node 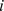; if the node receives more current than the sum of the outgoing currents, the node must increase in voltage to decrease the incoming currents and increase the outgoing currents, and vice versa. This updating process will continue until all nodes satisfy Kirchhoff's law.

Similar to Eq. (1), the current flowing from node 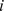 to node 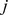 is given by Ohm's law for a given source 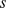 and universal sink (ground):
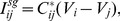
(4)where 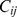 is the conductance of an ideal diode representing the edge from node 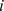 to node 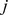:
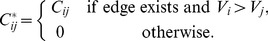
(5)The voltage of node 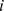 is determined by Kirchhoff's law that the sum of the currents one node supplies to its neighbors must be equal to the sum of the currents it receives.
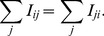
(6)When Eqs. (4), (5), and (6) are combined, the result can be expressed in terms of the potentials of the neighboring nodes:
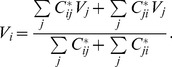
(7)Just as in the undirected case, the potential of each node is the weighted average potential of its neighbors. To compute the current flow, we need to enumerate all nodes, which takes the amount of 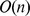 time. We consecutively update a node's voltage to the average voltage of its neighbors, according to Eq. (7). It takes the amount of 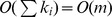 time to update the voltage in one loop, where 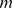 is the average degree of nodes. The updating process converges in a small number of steps, say, 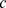. Thus the total computational time is 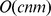 [15].

### Method of finding and distinguishing two types of key nodes

The overlapping and bridging nodes are located at conjunction positions, and the removal of these nodes will disable the interactions and communications among communities. As shown in Fig. 1, for example, node 16 and 24 are bridging nodes. They have edges with most of the nodes within their respective groups and a few edges that connect outside the groups. In the fields of community-network analysis and information dissemination, a bridging node controls information flow and diffusion; it has strong internal control within the community and strong connections among communities. Meanwhile, node 12 is an overlapping node between two communities. It connects the communities.

To identify the two types of nodes, the first task is to establish an index to distinguish them from the other nodes in a network. Considering an electrical-circuit network, nodes within a community are connected densely, and therefore their voltages may be similar to each other, while a large potential gap is present between two communities where the connecting edges are sparse and the local resistance is large. Thus, the current through the nodes or edges that connect the two communities can be significantly greater than the current through the nodes or edges within a community. Thus, a higher current value for a node indicates that it is more likely to be subject to the two types of nodes. As a second step, we note that overlapping nodes belong to more than one community and are usually associated with relatively denser connections to each community. We thus introduce the 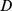 index to measure the imbalance of the current value on the edges of a node to separate the two types of nodes. The current-flow centrality 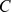 to measure the significance of a node, which takes into account the contributions of all paths to the node. For a given node, 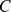 measures the current flow that passes through the node when a unit of current is injected into a source node and removed from a target node, averaged over all source-target pairs. Given a source 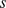 and a target 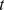, the absolute current flow through the edge(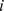,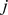) is given by Eq. (1). By Kirchhoff's law, the current that enters a node is equal to the current that leaves the node. Hence, the current flow through a node 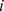 other than the source nodes and target nodes is half of the absolute flow on the edges incident to 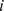:
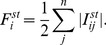
(8)Moreover, the current flows through both 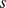 and 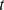 are set to fixed values. We give a precise definition of the current-flow centrality of a node:
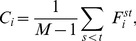
(9)where 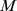 is the total number of source-target pairs. When extended to a directed network, there is little difference from the present case for an arbitrary node 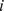 between the source node 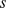 and the universal sink (ground). Due to the fact that
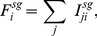
(10)we define the directed current-flow centrality 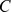 as:
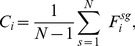
(11)where 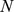 is the size of the network. When choosing pairs of nodes as the source and target each time, we obtain the current flow of the edge(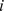,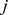). 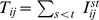 is the summed current flow through the edge(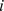,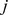) when the source and target nodes are changed. The 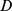 index of node 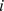 measures the difference between the max and median value of node 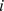:
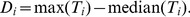
(12)Note that 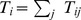, where the sum is over all the neighbors of node 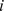. We normalize this 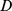 index by dividing by the maximum value of 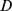. For a directed network, we merge each pair of in- and out-edges into one edge, and for node 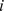, by adding the two current values, we can obtain the undirected and the directed 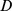 index.

## Results

### Performance on artificial networks

**Figure 4:**
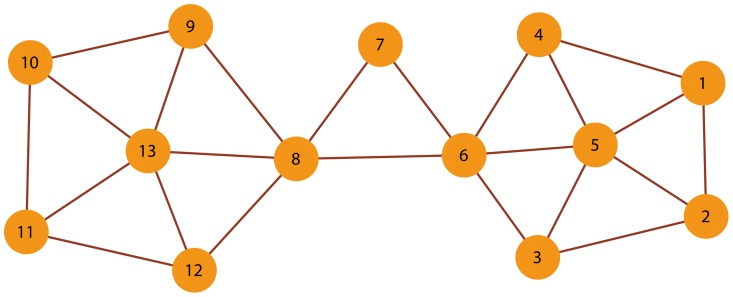
Example network with two groups. Each group contains six nodes, the central node 7 connects the two groups.

Prior to applying our method to real-world networks, we discuss the inherent limits of the betweenness-based method for inferring the two types of nodes. In principle, the index of betweenness centrality is exclusively determined by shortest paths but omitting the other longer paths, accounting for the missing of some critical nodes in some scenarios. In contrast, our current-flow-based method takes the sharing of current flow according to the conservation into account, giving rise to a more comprehensive characterization of the statues of nodes in the network with inapparent communities. Take a sample network as shown in Fig. 4 as an example. There are two communities, each of which consists of 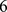 nodes. As table 1 shows, both of index rank nodes 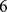 and 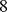 of highest, however, the betweenness fails to give a higher score to the topological central position node 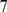 in this simple network. In contrast, our current-flow centrality 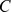 gives a relatively higher score of node 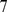. This explicitly indicates that the critical node 7 that bridges the two communities is missed by using the betweenness-centrality-based method.

**Table 1:** Centrality indices of the example sketched in Fig. 4.

Node Label	Current-flow centrality	Betweenness centrality
7	0.308	0
6,8	0.655	0.538
5,13	0.353	0.144
3,4,9,12	0.279	0.061
1,2,10,11	0.238	0.008

**Figure 5:**
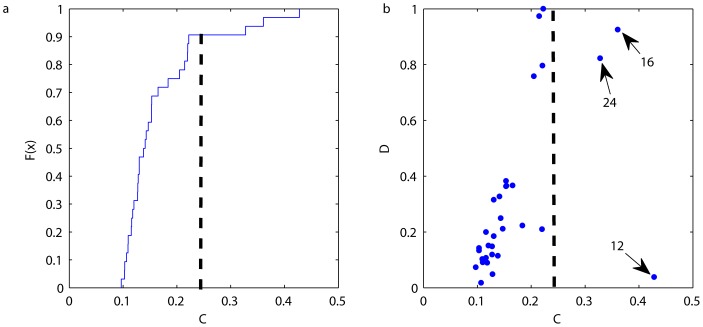
The usage of our method in the schematic network. (a) The cumulative distribution function of the *C* index. One can obtains a corresponding value of the *C* index when *F*(*C*) reaches 0.9. The dash line indicates the threshold. Nodes of higher value than the threshold are key nodes. (b) The scatter plot of indices *C* and *D*. A high *C* value and a low *D* value of 12 indicate that it could be considered as overlapping node, while 16 and 24 behave as bridging nodes.

To obtain a preliminary assessment of the underlying network characteristics identified by the indices 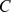 and 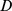, we apply them to an artificial network consisting of the nodes and edges shown in Fig. 1. The network is constructed by joining 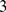 parts with bridging nodes and overlapping nodes. The small network size enables that any pattern present could be easily detected by visual inspection. As shown in Fig. 5(a), we artificially define the top 10% of nodes in terms of 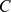 to be key nodes. In other words, the threshold of distinguishing the two types of key nodes from the other nodes is determined by the 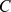 of top 10% of nodes. As shown in Fig. 5(b), the results reveal that the highest values of current flow occur in the nodes 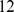, 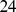, and 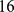. These nodes connect different communities of the network and plays important roles in the network. Despite their high values of 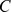, they differ in their 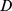 indices considerable. As stated before, a high 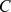 value and a low 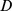 value of node 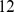 indicate that the node acts as an overlapping node that belongs to both the two communities that it connects. In contrast, 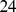 and 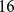 have high 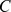 and high 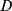 values simultaneously, indicating that they more likely to be bridging nodes.

**Figure 6:**
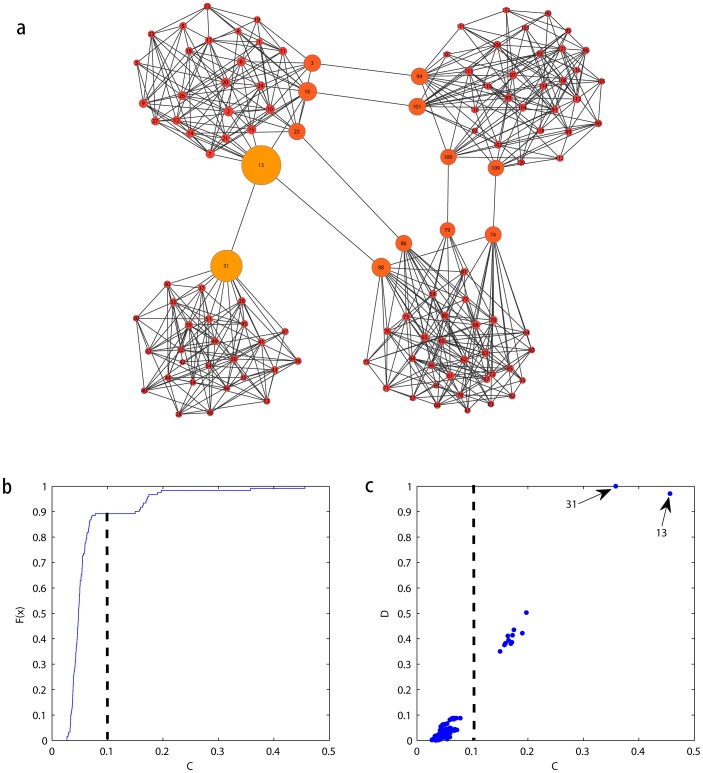
The usage of our method and in the LFR benchmark network. (a) The network is generated according to the rules of LFR benchmark. Nodes diameters indicate the current-flow centrality *C*, the color of each node is proportional to the index *D*. (b) The cumulative distribution function is used to identify the threshold of the *C* index. (c) the network can be separated by two categories according to the scatter plot, the upper right nodes can be considered as bridging nodes with high value of *C* and *D*. There are no overlapping nodes in this network.

We test our method on the LFR benchmark introduced by Lancichinetti et al. [18]. In the LFR benchmark, the node degrees follow a power-law distribution with the exponent 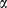, and the sizes of the communities follow another power-law distribution with then exponent 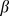. To ensure a clear community structure, we set 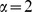, 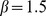, and 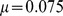. It can be intuitively understood that some nodes that connect two or more communities have large current values, corresponding to bridging nodes or overlapping nodes, as discussed before. Thus we need to introduce the 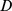 index to distinguish these two types of nodes by using the current-distribution information for each node. The results demonstrate that some nodes whose current values are significantly larger than those of other nodes may be regarded as the two types of key nodes. As shown in Fig. 6, the network can be well separated into two categories. The nodes at the upper right of the scatter plot have relatively high values of both 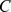 and 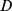, which indicates they have more internal edges than external edges. The nodes at the lower left are contained within communities and have few edges outside their communities. It can be claimed that there are no obvious overlapping nodes in this LFR benchmark, but it may contain some bridging nodes.

### Real-world networks

We test our method by using a number of real-world networks: the ZK network [19], the SFI network [20], and the C. elegans neural network [21]. All the data are available for the Open Access. In each case, we find that our method reliably detects the important nodes and ideally distinguishes the two types of nodes.

**Figure 7:**
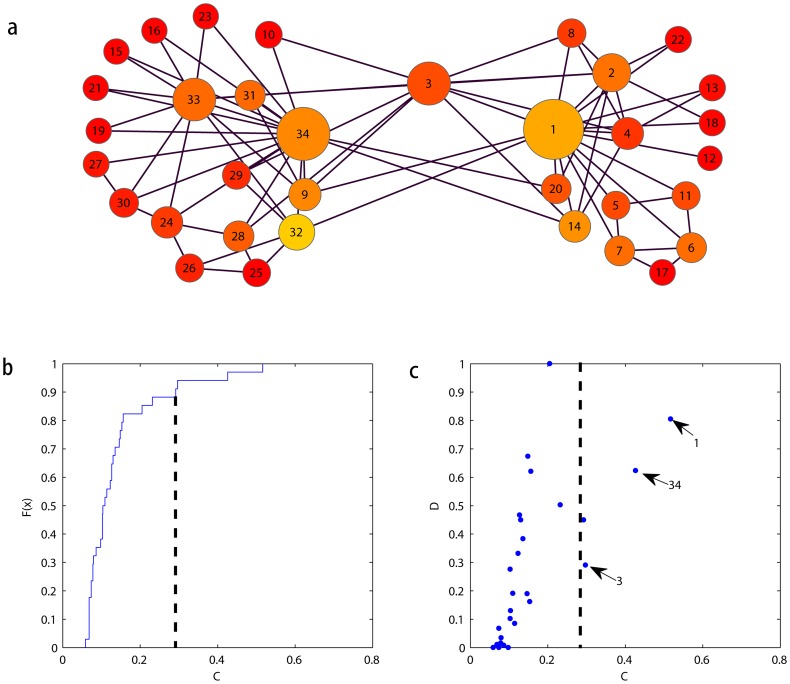
The usage of our method in ZK network. (a) The network consists of 34 nodes. The size of each node represents its *C* index value. The color of each node is proportional to its *D* index. (b) The cumulative distribution function of the *C* index and the threshold. In (c), nodes 1 and 34 act as bridging nodes because they have high value of *C* and *D* indices, in contrast, node 3 can be considered as overlapping node with a high *C* value and a low *D* value.

First, we consider ZK club network. In fact, Zachary observed 34 members of a karate club over 2 years. The nodes labeled as 1 and 34 correspond to the club instructor and the administrator, respectively. During the course of the study, a disagreement developed between the administrator of the club and the club's instructor, which ultimately led to the instructor leaving and starting a new club, taking approximately half of the original club's members with him. From the results shown in Fig. 7, nodes 1, 34, and 3 have the highest 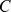 values and can be considered to be key nodes. Furthermore, node 3 is considered to be an overlapping node between the communities and displays a high value of current flow but a smaller 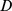 value. Our identified bridging node 3 is consistent with the overlapping nodes identified in ref [24], [25]. Nodes 1 and 34, which are known to be the administrator and instructor of the karate club, are more likely to be bridging nodes because they have high current values of 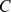 and high values of the 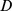, as discussed before. The visualization of the ZK network is shown in Fig. 7(a). The size of each node is proportional to the value of 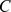. This visual perspective reveals that there are only a few nodes of large diameter, which means that few important nodes exist in this network. Additionally, a yellow color indicates a high value of 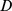. That is to say, large yellow nodes are more likely to be bridging nodes, while large red nodes are more likely to be overlapping nodes.

**Figure 8:**
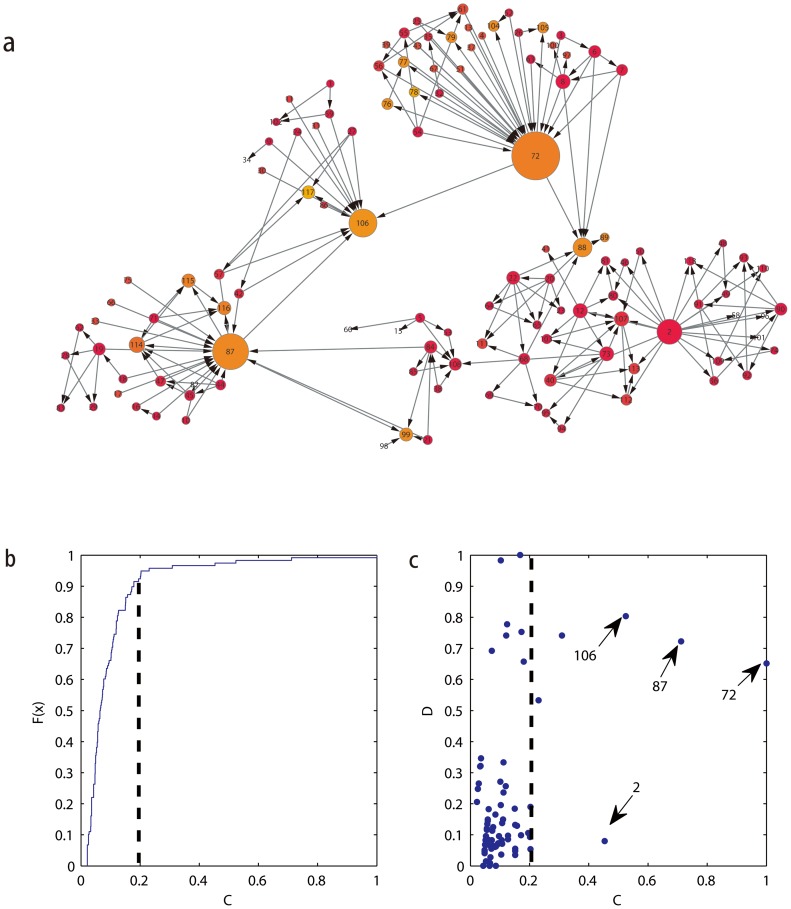
The usage of our method in the directed SFI scientist collaboration network. (a) Schematic of the SFI scientist collaboration network. Node diameters indicate the *C* index value, the color of each node is proportional to the index *D*. (b,c) The current-flow centrality *C* and index *D* for the directed SFI scientist collaboration network.

Applying the directed electrical-circuit network paradigm, we investigate the SFI scientific collaboration network. We convert it to be a directed network by randomly a direction to each of the edges. The result shown in Fig. 8(c) indicates that node 72, 87, 106, and 2 have high values of 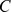, all these nodes act as connection points among communities. Due to the fact that node 106 has a high value of 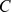 and a large value of the 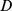, it can be considered to be a bridging node. In fact, from visual inspection of Fig. 8(a), we find that it has primarily inward-directed edges and only a few edges directed toward other communities, which means that this node transfers information that is received from the outside and spread in communities. Nodes 72 and 87 have similar characteristics, while node 2 behaves more like an overlapping node.

**Figure 9:**
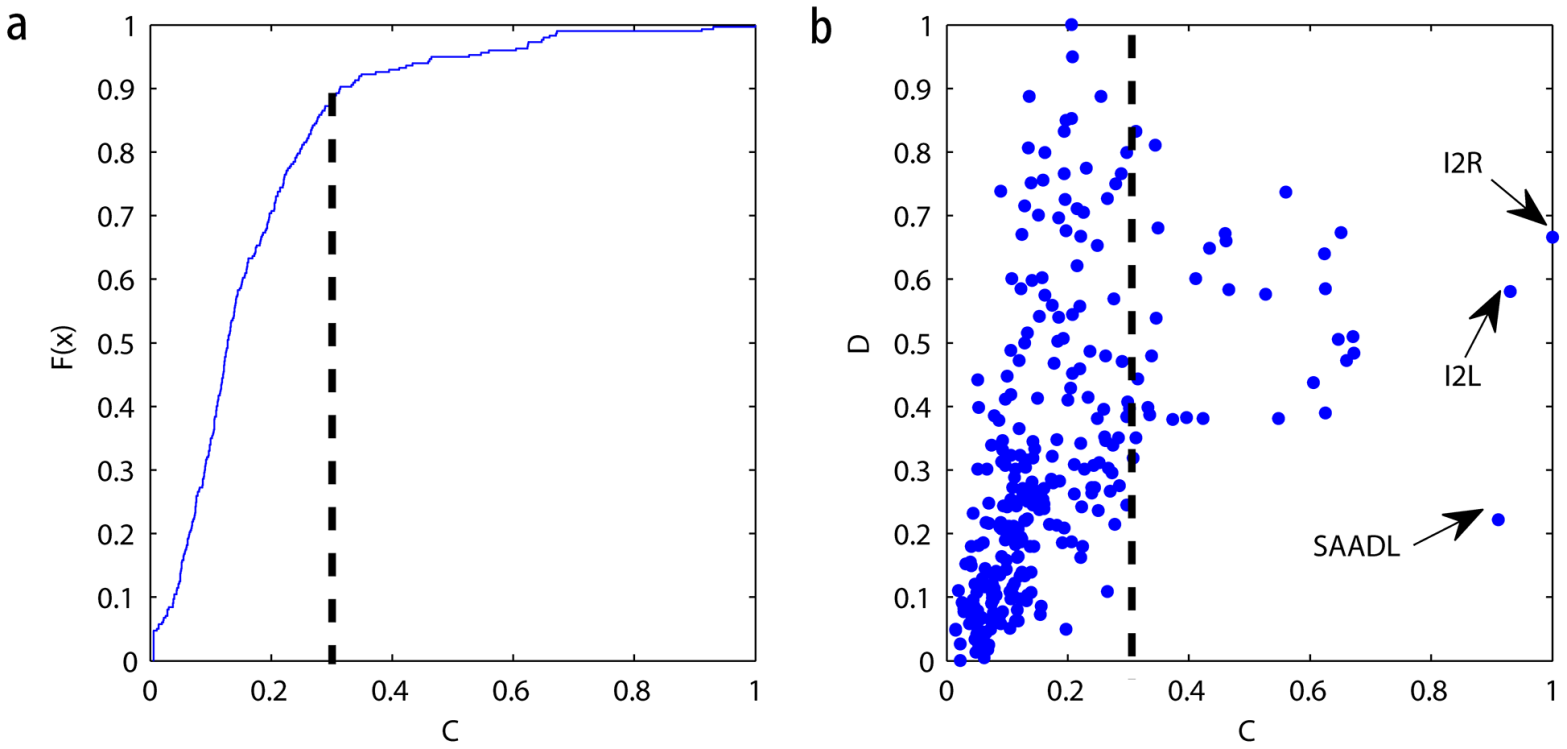
The usage of our method in the C. elegans directed neural network. (a,b) The current-flow centrality *C* and index *D* are calculated in the C. elegans neural network.

We also apply our method to another directed network: the C. elegans neural network [21]. The network contains 302 nodes and 2359 edges and is divided into 3 communities, with each node representing a neuron and each edge representing a synaptic connection between neurons. The C. elegans neural network is composed of sensory neurons, inter-neurons and motor neurons. The neurons with high centrality indices often have the most important functions, and all of them are inter-neurons. Applying our method to this network (see Fig. 9) demonstrates that a fairly large number of nodes have high values of 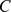, which indicates that there exists a significant proportion of neurons that are connected to different parts of the brain. Upon further investigation of these connection nodes, we find that the node named ‘SAADL’ has relatively low 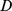 values, meaning that they are more likely to act as overlapping nodes rather than bridging nodes.

## Discussion

We have offered an electrical-circuit-based method to ascertain and distinguish overlapping and bridging nodes that play key roles in the communications and interactions among communities in complex networks without the need to partition all communities explicitly. The two types of critical nodes can be distinguished from the other nodes within communities by the relatively high current flow passing through them, as captured by the centrality of current flow. Further, the two types of nodes can be distinguished from each other via the imbalance of flows along their edges. In particular, the bridging edges of bridging nodes exhibit much high current flows than the other edges of the nodes. Whereas for the overlapping nodes, due to their dense connections to two communities and the absence of bridging edges, the current flows along their edges are relatively balanced. Thus the combination of the centrality of current flow passing through nodes and the imbalance of current flows along the edges of nodes offers a criterion for identifying the two types of nodes with high probability. In contrast, we have shown that the method for community partition based on the betweenness centrality cannot be used to address this problem. We have applied our method to a number of artificial and real networks with certain community structure, finding that the two types of nodes discovered by our method are in good agreement with the inspection of small visualized networks. Another advantage of our method is that it is available for both undirected and directed networks, accounting for its broad application scope in real situations.

Despite the advantages of our method compared to previously established methods in the literature, there are still some open questions pertaining to explicitly inferring overlapping and bridging nodes. For example, although our method is capable of finding these nodes with high probability, we continue to lack a reasonable threshold so as to exactly distinguish the two types of nodes. The challenge is rooted in the fact that there is only the measurement for the strength of communities rather than the exact definition of a community, accounting for the difficulty in exactly defining and recovering overlapping and bridging nodes. Nevertheless, our approach offers an alternative avenue for addressing the fundamental problem in complex networks and it is indeed effective and more efficient than existent methods in the literature based on the shortest paths and the betweenness centrality. Taken together, our approach could motivate further effort towards detecting the key nodes pertaining to ubiquitous community structures in complex networks.
